# Peroxisome proliferator-activated receptors (PPARs) and ovarian function – implications for regulating steroidogenesis, differentiation, and tissue remodeling

**DOI:** 10.1186/1477-7827-3-41

**Published:** 2005-08-30

**Authors:** Carolyn M Komar

**Affiliations:** 1 Department of Animal Science, Iowa State University, 2356 Kildee Hall, Ames, IA 50011, USA

## Abstract

The peroxisome proliferator-activated receptors (PPARs) are a family of transcription factors involved in varied and diverse processes such as steroidogenesis, angiogenesis, tissue remodeling, cell cycle, apoptosis, and lipid metabolism. These processes are critical for normal ovarian function, and all three PPAR family members – alpha, delta, and gamma, are expressed in the ovary. Most notably, the expression of PPARgamma is limited primarily to granulosa cells in developing follicles, and is regulated by luteinizing hormone (LH). Although much has been learned about the PPARs since their initial discovery, very little is known regarding their function in ovarian tissue. This review highlights what is known about the roles of PPARs in ovarian cells, and discusses potential mechanisms by which PPARs could influence ovarian function. Because PPARs are activated by drugs currently in clinical use (fibrates and thiazolidinediones), it is important to understand their role in the ovary, and how manipulation of their activity may impact ovarian physiology as well as ovarian pathology.

## Introduction

Peroxisome proliferator-activated receptors (PPARs) are a family of nuclear hormone receptors belonging to the steroid receptor superfamily. Issemann and Green identified the first PPAR in 1990 [1], and subsequently, two other family members were discovered. To date, PPARs have been identified in a variety of species from chickens [2] and fish [3], to humans (reviewed in [4,5]).

Although a great deal has been learned about PPARs since their discovery, very little is known regarding how these factors impact ovarian function. This review describes the expression of the PPARs in the ovary, and highlights the roles of these transcription factors that may affect ovarian biology. The influence of PPARs on polycystic ovary syndrome (PCOS) is not discussed in this review. There is a large body of literature on the use of thiazolidinediones, a class of drugs that activate PPARγ, in the treatment of women with PCOS. However, because these drugs can have direct effects on the ovary independent of activating PPARγ [6], and indirectly influence ovarian biology by lowering insulin levels, it is hard to discern PPARγ-dependent versus -independent effects. Therefore, this review focuses on the potential of PPARs to impact normal ovarian function and the development of ovarian tumors.

### PPARs

**Figure 1:**
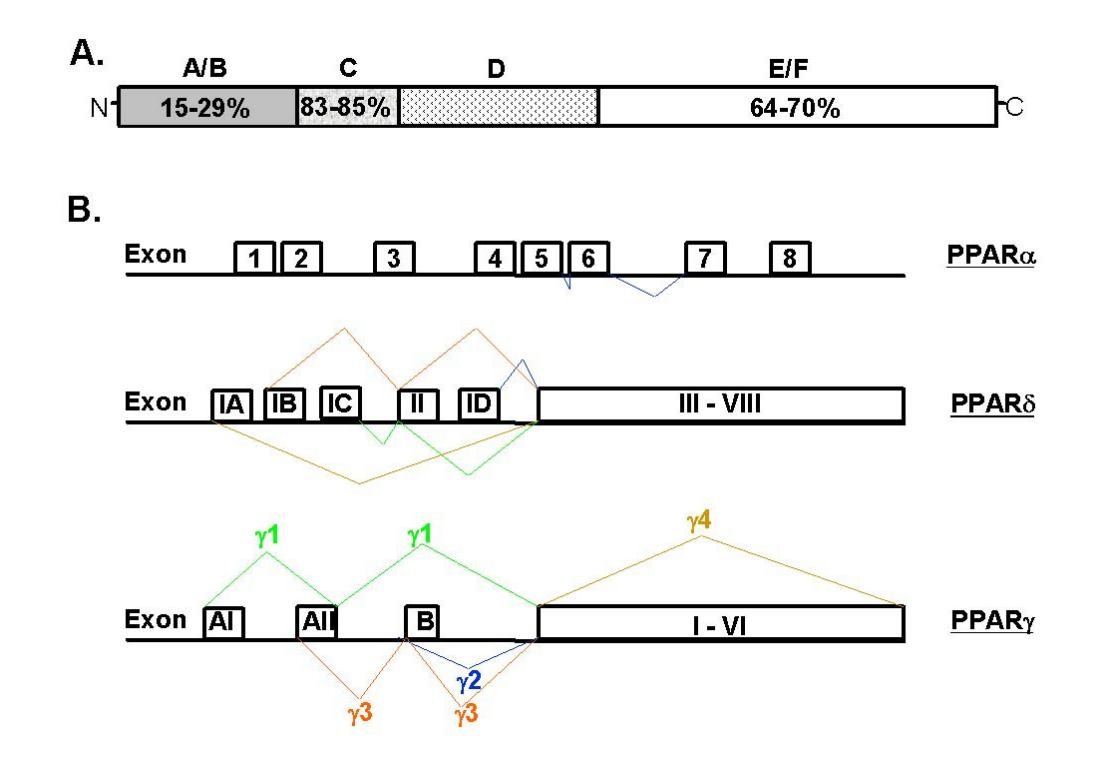
Structure, relationship and splice variants of the PPARs. A) Schematic diagram of the structure common to nuclear hormone receptors and the PPARs, indicating the relative similarities between the various regions of PPAR isotypes across species [4] [139] [140]. B) Schematic of the splice variants of the PPARs. Schematic of PPARα adapted from [7] [8] [141]. The diagram of PPARδ splice variants was adapted from [9]. Exons IA, IB, IC, ID, and 2 are non-coding. Regarding PPARγ splice variants, exons 1–6 are common to all PPARγ subtypes. PPARγ_1 _includes the untranslated exons A1 and A2, PPARγ_2 _contains the translated exon B, PPARγ_3 _contains the untranslated exon A2, PPARγ_4 _contains only exons 1–6 (adapted from [4] [10] [142]). Images not drawn to scale.

There are three PPAR family members: PPARα (NR1C1), PPARδ [NUC-1, fatty acid-activated receptor (FAAR), β, NR1C2], and PPARγ (NR1C3). The PPARs share a common structure with other steroid hormone receptors (Figure 1A). The N-terminal A/B domain is responsible for ligand-independent transactivation function (AF-1); the C domain contains the DNA-binding domain; the D domain – also called the hinge region, plays a role in receptor dimerization; and the C-terminal E/F domain contains the ligand binding domain (AF-2).

Each PPAR family member is transcribed from a specific gene. Alternative splicing and the use of different promoters give rise to different splice variants of each PPAR family member (Figure 1B). In addition to the full length mRNA for PPARα, in humans a splice variant has been identified which lacks the hinge region and the entire ligand binding domain [7,8]. This splice variant of PPARα can interfere with PPAR activity, and other nuclear receptors, by competing for coactivators [8]. Four spice variants for PPARs δ and γ have been identified. The splice variants for PPARδ give rise to one primary translation product [9]. PPARγ_1_, γ_3_, and γ_4 _yield the same protein product [10], whereas the protein encoded by PPARγ_2 _has an additional 30 (mouse) [11] or 28 (human) [12] amino acids in the N-terminus. Additional splice variants for PPARγ have been identified in monkey macrophages and adipocytes [13].

### Activity of PPARs

#### Ligand binding

There are a multitude of agents that activate the PPARs (Table 1). Many of these agents have well established roles in ovarian biology. For example, endogenous factors that have been shown to activate the PPARs that also impact ovarian function are fatty acids and prostaglandins, and exogenous activators include herbicides, industrial plasticizers, non-steroidal anti-inflammatory drugs (NSAIDs), fibrates (a class of drugs used to treat hyperlipidemia), thiazolidinediones (TZDs; hypoglycemia drugs), polycyclic aromatic hydrocarbons, organotin compounds, and traditional medicines [5,14-21]. An example of how these exogenous PPAR agonists impact the ovary is the inhibition of ovulation and 'reversible female infertility' caused by NSAIDs [22].

**Table 1:** Overview of ligands, both endogenous and exogenous, for the PPAR isotypes. Asterisks denote presence in the ovary, and/or reported affect on ovarian cells.

**Endogenous Ligands**	**Source**	**Specificity for PPAR isotype**	**Reference**
Polyunsaturated fatty acids*	Diet Metabolism	PPARα>PPARδ>>PPARγ	[25]; reviewed in [5]
Eicosanoids*	Inflammation	PPARα, PPARδ, PPARγ	[25]
8-HETE	Metabolism	PPARα	
PGJ_2_		PPARγ>>>PPARα>PPARδ	[17] [26]
PGA_1_		PPARδ>>PPARα,PPARγ	[17] [25]
PGI_2_		PPARδ	reviewed in [16]
Leukotriene B_4_		PPARα	[23] [25]
Lysophosphatidic acid*	Metabolism	PPARγ	[18]
Oxidized LDL	Metabolism	PPARγ	reviewed in [29]
			
**Exogenous Ligands**	**Source**	**Specificity for PPAR isotype**	**Reference**

Herbicides/fungicides	Environment	PPARγ	[19]; reported in [1]
Plasticizers*	Environment Industry		[137]; reviewed in [15]
NSAIDS	Pharmaceutical	PPARγ>PPARα>>PPARδ	[20] [28]
Fibrates*	Pharmaceutical	PPARα>>>PPARγ	[25]
Polycyclic aromatic hydrocarbons	Environment	PPARα, PPARδ	[21]
Herbal/plant compounds	Traditional medicine	PPARα, PPARγ>PPARδ	reviewed in [14]
Genistein*	Plants	PPARγ	[138]
Thiazolidinediones*	Pharmaceutical	PPARγ	[23] [55]

There is some specificity observed between ligands and the PPAR subtypes. For example, fibrates (i. e. WY-14,643, clofibrate) show a high affinity for PPARα, but at higher concentrations can also activate PPARγ [4]. The thiazolidinediones (troglitazone, ciglitazone, pioglitazone, rosiglitazone) selectively activate PPARγ [4,23]. Long chain fatty acids, particularly polyunsaturated fatty acids, preferentially activate PPARα [24], but are also capable of activating PPARδ and PPARγ [5,23,25]. Prostaglandins activate all PPAR family members, with PGA_1 _and 15-deoxy-Δ^12,14^-prostaglandin J_2 _(PGJ_2_) preferentially activating PPARδ and PPARγ, respectively [5,25,26]. Prostacyclin and its analogue, carbaprostacyclin, also binds to PPARδ (reviewed by [16,27]). Hydroxyeicosapentaenoic acids and leukotriene B4 are activators of PPARα [5,25]. Interestingly, indomethacin and other NSAIDs that inhibit the production of prostaglandins, are also able to activate PPARα and PPARγ [28]. Oxidized products of LDL (9-HODE and 13-HODE) are ligands for PPARγ (see [4]).)[29] for a review). Structural and amino acid differences in the binding pocket of the PPAR isoforms contribute to selectivity for ligand binding [30].

#### DNA binding

**Figure 2:**
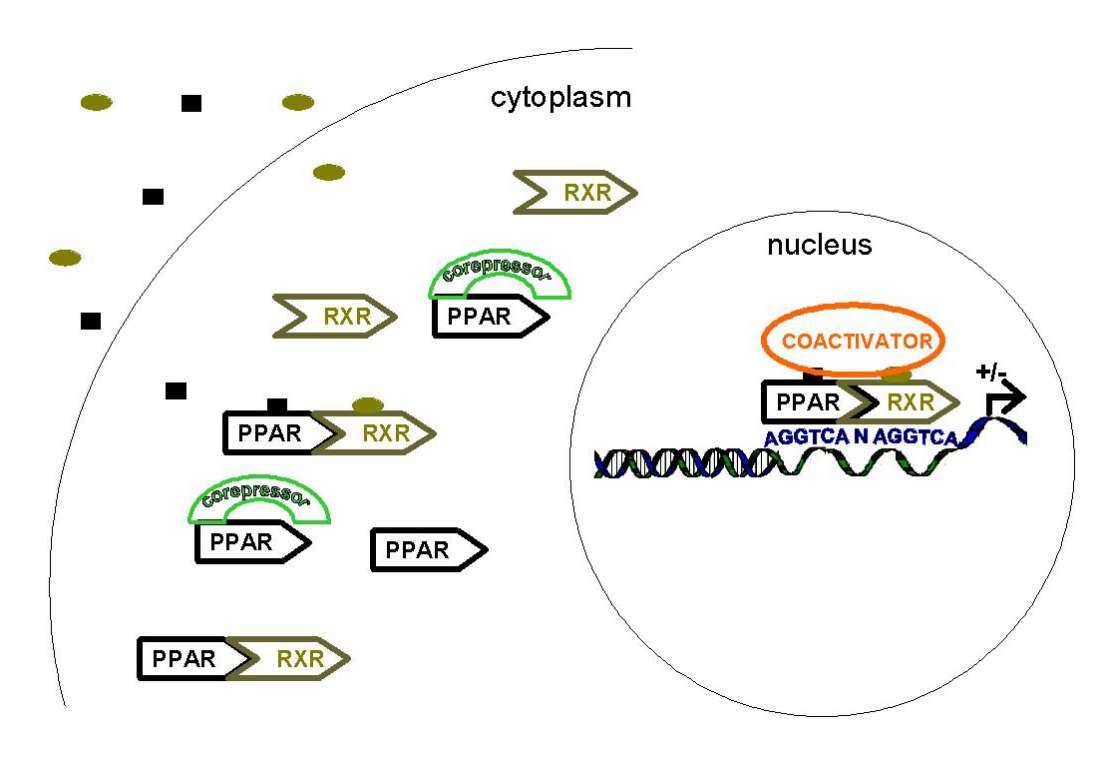
Mechanism of action of PPARs. PPARs heterodimerize with RXRs both in the presence and absence of ligand. After ligand binding, PPARs undergo conformational change resulting in dissociation of corepressors, and the binding of coactivators. PPAR/RXR heterodimers bind to a DR1 sequence in the promoter region of target genes (see text for details).

PPARs heterodimerize with 9, *cis*-retinoic acid receptors (RXRs) (Figure 2). PPAR interaction with RXRs can occur in the absence and/or presence of ligand [31]. The heterodimer binds to a short sequence of DNA, a PPAR response element (PPRE), present in the promoter regions of target genes. The PPRE is a direct repeat of the sequence AGGTCA, separated by one nucleotide (a DR1 sequence; reviewed in [4,5]). In addition to the PPRE, the 5' flanking region has been shown to be important for PPAR binding to DNA, especially PPARα binding. The binding affinity of the PPAR/RXR heterodimer is greatly enhanced if the nucleotide between the two hexamers is an adenine, and when there is an AA/TCT sequence 5' of the PPRE (reviewed in [4,5,32]). These DNA features result in a polarity to the bound heterodimer; PPAR binds to the upstream hexamer while RXR interacts with the lower, 3' hexamer [5,32]. The integrity of the 5' sequence offers selectivity in binding for the PPAR isotypes.

#### Cofactors

Similar to other steroid hormone receptors, there are coactivators and corepressors that associate with the PPARs. Corepressors, such as nuclear receptor corepressor (NCoR) and silencing mediator for retinoid- and thyroid-hormone receptors (SMRT), dissociate from the receptor upon ligand binding (reported in [4,33]). The conformational change that occurs upon ligand binding also facilitates the recruitment of coactivators. Two coactivators that have histone acetyltransferase activity, steroid receptor coactivator-1 (SRC-1) and CREB binding protein/p300 (CBP), can bind to PPARs in a ligand-dependent manner. The latter coactivators can also interact with PPARs in a ligand-independent manner, but only transiently (reviewed in [4]). RIP140, ARA70, and members of the DRIP/TRAP family of coactivators also bind to PPARs (see [34] for a review). Other coactivators that have been identified to interact with PPARs are: PPAR interacting protein [33], PPARγ coactivator-1 (reviewed in [35]), and PPAR binding protein (PBP; [36]). Although these coactivators also bind other steroid receptors, deletion of the PBP gene in mice results in embryonic lethality due to placental insufficiency [37], the same results seen in PPARγ null mutants [38]. These findings are consistent with the hypothesis that PBP is a required factor for PPARγ transcriptional activity. The regulated expression of these various corepressors and coactivators and their concentrations in tissues also offers selectivity in transcriptional regulation by the PPAR isotypes.

A recent intriguing finding is that the association of corepressors with PPARδ can inhibit the activity of PPARs α and γ. Shi *et al*. (2002) demonstrated that PPARδ repressed PPARα and γ-mediated gene transcription. This repressive activity of PPARδ involved DNA binding and association with the corepressor SMRT [39]. The authors of this study concluded that the levels of each PPAR isotype, as well as the ratio of PPARs α and γ to PPARδ in a particular tissue influences the activity of each isotype.

#### Post-translational modifications

The activity of PPARs are modified not only by ligand binding, but also by phosphorylation, nitration, and ubiquitination. Phosphorylation sites have been identified on both PPARs α and γ. The impact of phosphorylation on the activity of PPARs depends on: 1) the residue being phosphorylated, and 2) the kinase cascade that was activated (reviewed in [40]). A modification of PPARγ that influences its activity is nitration of tyrosine residues. Shibuya *et al*. (2002) demonstrated that nitration of tyrosine residues in PPARγ inhibited the translocation of PPARγ from the cytosol to the nucleus [41], thus reducing its potential to influence gene transcription. PPARs can also be ubiquitinated. Ligand binding to PPARγ induces ubiquitination of the receptor [42], and therefore its degradation. In contrast, ligand binding to PPARα stabilizes the receptor by decreasing its rate of ubiquitination [43,44].

### Expression and functions of PPARs

The tissue distribution of mRNA differs among the individual PPAR family members. PPARα is an important player in regulating fatty acid metabolism [4,45], and it is expressed at relatively high levels in the liver, small intestine, kidney, heart, and brown adipose tissue [46,47]. It has also been demonstrated to play a role in inflammation (reviewed in [5,35,48]). PPARδ is ubiquitously expressed with highest levels of expression seen in the liver, kidney, and brown adipose tissue in the mouse [4,46,47,49]. A study of PPARδ null mice illustrated that this PPAR subtype is involved in development, lipid metabolism, proliferation of epidermal cells, and myelination of nerves [50]. PPARδ also plays a role in wound healing (reviewed in [51]), embryonic implantation [52,53], and adaptive responses to exercise in skeletal muscle (reviewed in [54]). The expression of the various PPARγ isoforms shows tissue specificity. PPARγ_1 _is the most widely expressed and is found in most tissues [4,49,55]. PPARγ_2 _is localized primarily to adipocytes, and PPARγ_3 _is also found in adipocytes, as well as colonic epithelium, and macrophages [46,56]. The distribution of PPARγ_4 _is unclear because it cannot be discriminated from PPARγ_1 _or γ_3 _due to the similarity between them [10]. PPARγ has been shown to be an adipocyte differentiation factor (reviewed in [57,58]), and also plays a role in glucose homeostasis, the cell cycle, carcinogenesis, lipid metabolism, and inflammation (reviewed in [35,59,60]). It has been suggested that PPARs mediate dietary regulation of gene expression due to the fact that various metabolic and nutritional agents can activate these transcription factors.

### PPARs and ovarian function

#### Expression and activity

**Figure 3:**
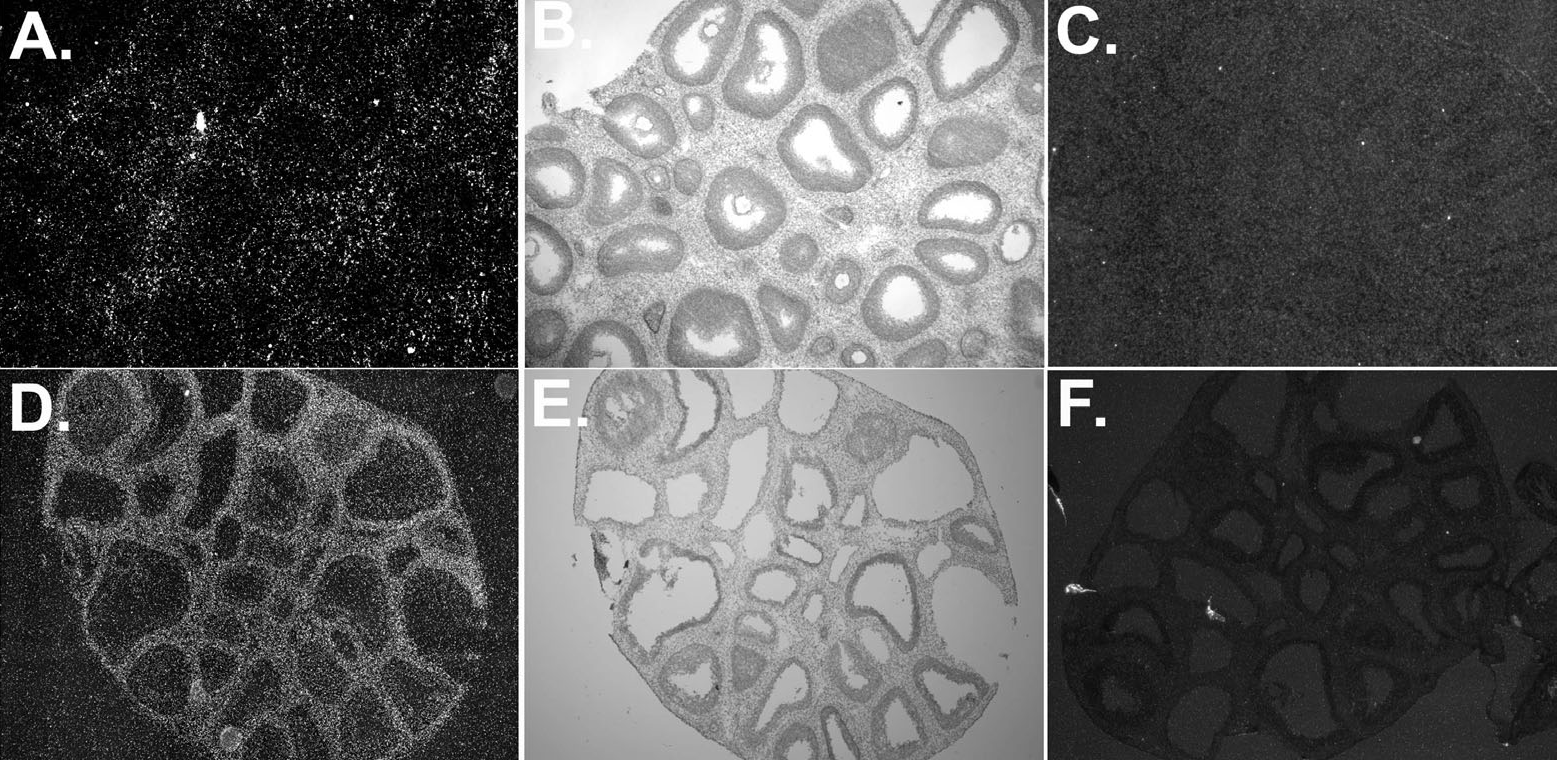
Localization of mRNAs corresponding to PPARα (A, B, C) and PPARδ (D, E, F) in ovarian tissue collected from immature rats 48 hours post-eCG. Tissue sections (8 μm) were hybridized with ^35^S-labled antisense (A, D) and sense (C, F) riboprobes for each respective PPAR isotype. Figures originally published in [62].

All three PPAR subtypes have been detected in ovarian tissue. In the rat ovary, the expression of mRNA for PPARα is found primarily in the theca and stroma, whereas mRNA for PPARδ is found throughout the ovary (Figure 3). The expression of these two PPAR isotypes remains steady throughout follicular development and the ovarian cycle in the rat [61,62].

**Figure 4:**
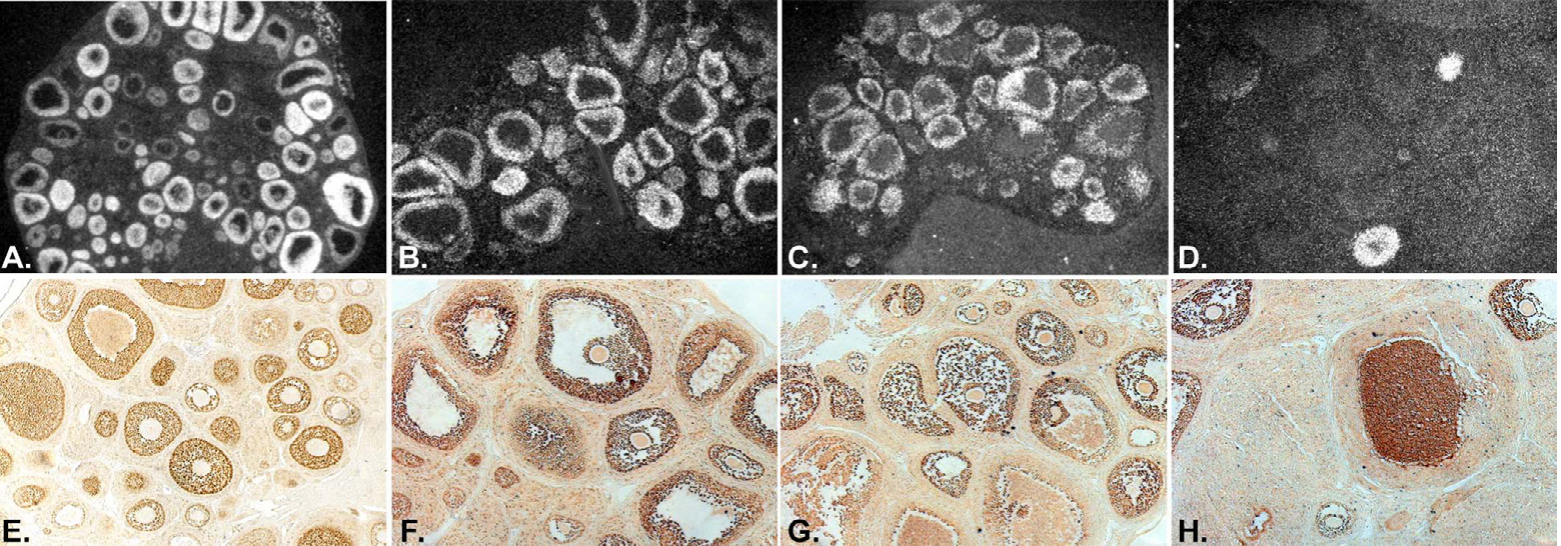
Localization of mRNA and protein corresponding to PPARγ in ovarian tissue collected from immature rats 0 (A, E) and 48 (B, F) hours post-eCG, and 4 (C, G) and 24 hours (D, H) post-hCG. Frozen tissue sections (8 μm) were hybridized with an antisense riboprobe corresponding to PPARγ. Figures A – D originally published in [71]. Protein corresponding to PPARγ, identified by the brown reaction product, was localized in 4% paraformaldehyde-fixed, paraffin embedded tissue using an anti-PPARγ antibody (Santa Cruz).

PPARγ has been more extensively studied in ovarian tissue than the other two family members. It has been detected in the mouse [63], rat [49,62], pig [64], sheep [65], cow [66,67], and human [55] ovary. Using RT-PCR, PPARγ was detected in granulosa cells collected during oocyte aspiration from women undergoing treatment for *in vitro *fertilization [68], and in porcine theca and granulosa cells [64]. This PPAR isotype has also been reported to be in oocytes from cattle [69], zebrafish [3], and *Xenopus laevis *(trace amounts; [70]). In cycling rats and sheep, the expression of PPARγ is restricted primarily to granulosa cells in developing follicles [61,62,65]. However, unlike the steady expression of PPARs α and δ, the expression of PPARγ is down-regulated in response to the LH surge (Figure 4) [62,65]. The expression of PPARγ decreases only in follicles that have responded to the LH surge [71]. In the rat, expression of PPARgamma is low in newly forming luteal tissue, and higher in luteal tissue present from previous ovulations [61]. Because PPARγ is primarily expressed in granulosa cells, it may influence development of these cells and their ability to support normal oocyte maturation. PPARγ could also potentially affect somatic cell/oocyte communication not only by impacting granulosa cell develpment, but by direct effects on the oocyte. Disrupting the expression of PPARγ in the ovary therefore, could potentially affect oocyte developmental competence.

Results from a study by Cui *et al. *(2002) indicate that PPARγ plays an important role in normal ovarian function. Using *cre/loxP *technology, the expression of PPARγ was disrupted in the ovary, rendering 1/3 of the females sterile, and the remaining females sub-fertile [63]. Females that were sub-fertile took longer to conceive and had smaller litters. There were no differences found in the number of primordial, primary, or preantral/antral follicles, size of copora lutea, or response to exogenous gonadotropins between control animals and those with PPARγ disrupted in the ovary. On the day of estrus, levels of progesterone in animals with PPARγ disrupted in the ovary were half that found in controls. However, the differences in circulating progesterone were not significantly different between the two groups, most likely due to the small sample size (n = 4/group). Implantation sites (6) were only observed in the uterus of one of three females examined with PPARγ disrupted in the ovary, compared with 5 and 7 implantation sites observed in two control females, respectively. Because the expression of PPARγ was not disrupted in the uterus of these transgenic females, the lesion responsible for the sub- and infertility most likely lies within the ovary. The authors concluded that "...ovarian function might not be sufficient to induce implantation" [63]. The insufficient ovarian function may relate to the ability of the corpus luteum to produce enough progesterone, or produce enough progesterone in a timely manner, to support the establishment of pregnancy. In addition, estradiol production by the ovary around day 4 post-coitum is also an important player in preparing the uterus for implantation. Impaired production of estradiol by ovarian cells in the transgenic females during this critical period may also lead to reduced implantation. Although not tested in this study, the competence of the oocyte to undergo fertilization and support embryonic development might also be compromised in these genetically altered mice. Further study into the role of PPARγ in ovarian steroidogenesis and somatic cell/oocyte interactions is needed to determine the cause of the fertility problems in females with reduced ovarian PPARγ expression.

Additional studies have shown that endogenous PPARγ is active in the ovary. Granulosa cells from rats and sheep were transiently transfected with reporter constructs whose expression was driven by PPREs. Both in the absence and presence of agonists for PPARγ, there was an increase in reporter activity [65,72]. PPARγ in rat granulosa cells was also shown to bind DNA [73]. These findings demonstrate that PPARγ is functional in granulosa cells, and that endogenous ligand is also present within these cells.

#### Regulation of Steroidogenesis

One way PPARs may influence ovarian function is by modifying the ability of estradiol to elicit cellular responses. PPARs are able to bind to estrogen response elements – EREs, [74,75], and can act as competitive inhibitors [74]. PPARγ can also stimulate ubiquitination of estrogen receptor α, leading to its degradation [76].

The synthesis and metabolism of estradiol is also affected by the PPARs. PPARγ can inhibit the expression of aromatase, the rate limiting enzyme for the conversion of androgens to estradiol by disrupting the interaction of NF-κB with the aromatase promoter II [77]. Activation of PPARα decreased the expression and activity of aromatase in granulosa cells [78,79]. In cultured human granulosa-luteal cells [68], and granulosa cells from eCG-primed immature rats [78], activation of PPARγ reduced the expression of aromatase. PPARγ was also shown to partially mediate the suppressive effects of phthalates on ovarian estradiol production [78]. However, using a different strain of rat and culture model, agonists of PPARγ were shown to increase estradiol secretion by granulosa cells collected from gonadotropin-primed immature rats [62]. Reduced levels of aromatase in granulosa cells after activation of PPARγ was also reportedly due to increased turnover in conjunction with decreased transcription [80]. We reported previously that there was no correlation between the expression of mRNAs for PPARγ and aromatase in granulosa cells during folliculogenesis or the periovulatory period [71]. PPARs may also limit the synthesis of estradiol by reducing production of androgenic precursors by theca cells. PPARγ is expressed in the theca [61,64], primarily in the theca externa and in an inconsistent pattern [61]. Both endogenous (PGJ_2_) and exogenous (troglitazone) agonists of PPARγ reduced basal and LH-stimulated thecal androgen production *in vitro *[64,81]. One study reported that troglitazone increased mRNA for CYP17, but not the corresponding protein [64], whereas a second study showed no effect of the PPARγ agonists on mRNA for CYP17, but a decrease in its phosphorylation [81]. In both granulosa [78] and liver cells [82], agonists of PPARα stimulated the expression of 17β-hydroxysteroid dehydrogenase type IV, an enzyme that oxidizes estradiol into the less active estrone. The expression of PPARα in granulosa cells is very low [61,62] and therefore may be unlikely to modify estradiol metabolism under normal physiological conditions. Taken together, these data indicate that PPARs are able to influence estradiol production, and that age and the endocrine environment may influence how these transcription factors impact ovarian steroidogenesis.

The activation of PPARγ can also influence progesterone production by ovarian cells. In cultured human granulosa cells, activators of PPARγ inhibited basal and gonadotropin-stimulated progesterone production [83]. However, activators of PPARγ stimulated progesterone secretion by granulosa cells obtained from eCG-primed immature rats [62]. When porcine theca cells were treated with synthetic and natural ligands for PPARγ, progesterone production increased [64]. Progesterone production by bovine luteal cells treated with the endogenous ligand for PPARγ, PGJ_2_, increased progesterone production over a 24 hour culture period [67]. Our previous work has shown that there is an inverse relationship between the expression of mRNA for PPARγ and P450 side chain cleaveage, the rate limiting enzyme in progesterone synthesis, in granulosa cells and luteal tissue from naturally cycling and gonadotropin-treated rats [71,84]. Therefore, the effect of PPARγ on progesterone production may depend on the cell type, stage of differentiation, stage of the cycle, and/or the species studied.

#### Tissue Remodeling

PPARs regulate the expression and activity of proteases involved in tissue remodeling and angiogenesis which are critical processes for follicular and luteal development. Plasminogen activators (PA) and matrix metalloproteinases (MMPs) are proteolytic enzymes involved in ovarian tissue remodeling and angiogenesis [85-87]. Activation of PPARα and PPARγ decreases MMP-9 expression and its activity [88-91]. The promoters for MMP-3 [92] and MMP-9 [93] contain a PPRE, indicating that transcription of these proteases is likely directly regulated by PPARs. PPARγ activation can also reduce expression of MMP-13 and MMP-1 by interfering with AP-1 activation [94-96]. PPARγ negatively affects plasminogen activator by inhibiting its expression [97] and increasing the expression of plasminogen activator inhibitor-1 [97,98]. However, there are also reports of troglitazone treatment reducing the expression of plasminogen activator inhibitor-1 [99,100]. These findings indicate that the PPARs are capable of modulating the balance of proteolytic enzymes and their inhibitors, thereby altering tissue remodeling events. Whether PPARs regulate these processes in ovarian cells, particularly at the time of ovulation when MMP and PA activities must be tightly regulated, is an important area of investigation.

Along with the proteases, vascular endothelial growth factor (VEGF) and its receptors (Flt-1, -2) are important players in new blood vessel formation in the ovary [101,102]. The activation of PPARγ with PGJ_2 _inhibited the expression of Flt-1 and Flt-2 in human umbilical vein endothelial cells [97]. Activation of PPARγ with its endogenous and exogenous ligands has also been shown to inhibit VEGF-stimulated endothelial cell proliferation and migration (reviewed in [103]). However, Yamakawa *et al. *(2000) reported that activating PPARγ in vascular smooth muscle cells results in an increase in the expression of VEGF [104]. Therefore, the effect of PPARγ on angiogenesis may depend on agonist used, experimental model, and/or cellular differences in cofactor availability [103]. Besides its effects on angiogenesis, PPARγ may influence the ovarian vasculature by its ability to regulate endothelin-1 (ET-1) and nitric oxide synthase (NOS). ET-1 is a potent vasoconstrictor and recent studies have shown that it is also an important player in ovarian physiology, especially luteal function (reviewed in [105]). NOS synthesizes nitric oxide, a vasodilator, from arginine. Nitric oxide has been implicated as a player in luteolysis [106], ovarian cyclicity [107], ovulation [107-109], oocyte maturation [108], and follicular development [110,111]. PPARγ decreases the secretion of ET-1 from endothelial cells [112], and also inhibits the expression of NOS in macrophages [90] and vascular smooth muscle cells [113].

The ability of PPARs to affect tissue remodeling could alter folliculogenesis and luteal development, and impact ovulation. Ovarian tissue is constantly changing to accommodate the dynamic geometry of growing follicles which increase in size exponentially from the primordial to preovulatory stage. For successful release of the oocyte at ovulation, the granulosa cell layer, follicular basement membrane, theca interna and externa, ovarian stroma, tunica albuginea, and surface epithelium need to be traversed. In addition, the tissue remodeling involved in developing the increased vasculature required to support follicular development and luteal formation requires protease activity. The ability of PPARs to regulate the expression of proteases and angiogenic factors, and the fact that they are expressed in the ovary and in the case of PPARγ, modulated during the periovulatory period encompassing ovulation and luteal formation, warrant further study into how the PPARs may influence these aspects of ovarian biology.

PPARs are important mediators of inflammatory responses (reviewed in [27,114-116]). The process of ovulation has been likened to an inflammatory response [117] and prostaglandins, major regulators of inflammation, have well documented roles in ovulation as well as luteal function (see [118] for a review). The rate-limiting enzyme in prostaglandin production is cyclooxygenase-2 (COX-2). The promoter region of COX-2 contains a response element for the PPARs [119], indicating that PPARs can directly influence transcription of this gene. However, there are reports of PPARγ both stimulating [119] and inhibiting [120,121] the expression of COX-2. In rat granulosa cells, the expression of COX-2 is stimulated within 4 hours of the ovulatory gonadotropin surge [122], however, PPARγ is significantly reduced in this same time frame [62]. This inverse relationship between the expression of PPARγ and COX-2 has also been observed in the placenta [123]. The variability in reported effects of PPARγ on COX-2 expression could result from: 1) the use of different cell-types, 2) transfection with COX-2 promoter constructs that did [119] or did not [124] contain the PPRE, 3) the ability of PPARs to influence COX-2 expression by binding to its promoter region, and/or 4) by PPARγ interfering with activation of NF-κB [121]. The periovulatory expression pattern of PPARγ suggests it plays an inhibitory role in COX-2 expression in ovarian cells *in vivo*.

Not only can PPARs regulate COX-2 expression, but as discussed earlier, prostaglandins themselves are endogenous ligands that can activate PPARs. In addition, PGF_2α _can activate kinase cascades resulting in the phosphorylation of PPARγ and inhibiting its activity [125]. Cumulatively, these findings imply that there is a cyclic relationship between the presence of prostaglandins, activation and/or inhibition of PPARs and feedback to the prostaglandin synthesizing enzyme – COX-2.

### PPARs, cell cycle regulation, and ovarian tumors

The minority of follicles which successfully develop to the preovulatory stage must balance cellular proliferation as well as escape from programmed cell death, or apoptosis. PPARs have well documented roles in apoptosis as well as cell cycle control (reviewed in [35,60,126,127]). For example, the gene encoding bcl-2, an anti-apoptotic factor, has a PPRE, and transfection of PPARγ increased bcl-2 protein and mRNA [128]. However, administration of troglitazone to cultured rat granulosa cells decreased levels of mRNA for bcl-2 and stimulated apoptosis [73]. Froment *et al. *(2003) also reported that treating granulosa cells from sheep with a PPARγ agonist decreased granulosa cell proliferation [65]. One cell cycle regulator, cyclin D2, shares a similar profile of expression to that of PPARγ, however, there are conflicting reports of how activation of PPARγ affects cyclin D2. In human leukemic cells, activation of PPARγ by troglitazone or PGJ_2 _resulted in a decline in mRNA and protein for cyclin D2 [129]. Like PPARγ, cyclin D2 is expressed in granulosa cells of developing follicles and down-regulated within 4 hours of the LH surge, but only in follicles that responded to the gonadotropin surge [130]. However, administration of troglitazone to cultured rat granulosa cells had no effect on cyclin D2 [72]. Thus, more work investigating the role of PPARγ in granulosa cell cycle progression is needed to address the apparent dichotomy of PPARγ inhibiting cell proliferation yet being expressed at a high level in developing follicles.

PPARγ is expressed in cells from a granulosa cell tumor [131], and up-regulated in epithelial ovarian carcinomas [132]. Interestingly, the expression of PPARγ was higher in malignant tissues than in benign tumors [132]. A study investigating the relationship between the expression of PPARγ and COX-2 in human epithelial ovarian tumors reported that there was an inverse relationship between the expression of these two factors [133]. Because over-expression of COX-2 is associated with various cancers ([133] and references therein), the authors of this latter study concluded that PPARγ and its activation may be beneficial in halting the progression of ovarian tumors.

Genetic susceptibility for developing ovarian and breast cancer is linked to the BRCA1 gene. BRCA1 is a tumor suppressor, and has been shown to be down-regulated in many cases of sporadic ovarian cancer. A study by Pignatelli *et al. *(2003) has shown that there is a PPRE in the promoter region for the gene encoding BRCA1, and both synthetic and endogenous ligands for PPARγ increase levels of BRCA1 in MCF-7 breast cancer cells [134]. Support for PPARγ playing a role in susceptibility to ovarian cancer *in vivo *comes from a study of mice heterozygous for PPARγ. Both heterozygous (PPARγ^+/-^) and wildtype mice were treated with the carcinogen 9, 10-dimethyl-1,2-benzanthracene (7, 12-dimethylbenz[*a*]anthracene). PPARγ^+/- ^mice had increased occurrences of ovarian granulosa cell carcinomas compared with wildtype littermates and the tumors that developed in PPARγ^+/- ^mice were more advanced than those formed in wildtype animals [135]. Taken together, these data strongly indicate that PPARγ may provide a protective effect against the development of chemically induced, as well as sporadic ovarian cancer.

PPARγ is not the only PPAR isotype with differential expression observed in ovarian carcinomas. In a subgroup of ovarian endometrioid adenocarcinomas associated with deregulated β-catenin, the expression of PPARδ was significantly elevated [136]. Because of the potential for PPARs to influence the cell cycle and apoptosis, de- or misregulation of these factors may be one mechanism associated with transformation of healthy cells into tumor cells.

### Future directions

The clinical use of drugs that activate the PPARs (fibrates and thiazolidinediones) and their ability to be activated by dietary agents warrents further investigation into the role of these transcription factors regulating ovarian gene expression. The inverse expression of PPARγ and P450 side-chain cleavage, and reduction in expression of PPARγ in response to LH, suggests that down-regulation of this transcription factor is important for ovulation and luteinization of follicular cells. Investigating the impact of PPARγ on the periovulatory period could be done by overexpressing PPARγ in granulosa cells, or altering PPARγ to prevent its down-regulation by LH and determining how this affects ovulation and the differentiation of follicular cells into luteal cells. Such information would elucidate mechanisms involved in the terminal differentiation of follicular cells and potentially what may go wrong leading to sub-functional corpora lutea. Investigating the influence of PPARγ on oocyte and follicular cell growth and maturation is also needed due to its high expression in granulosa cells of developing follicles and the sub- and infertility observed in mice with PPARγ disrupted in the ovary. The use of transgenic mice lacking PPARγ in the ovary and siRNA or similar technologies to reduce expression of PPARγ in cultured cells coupled with microarray and/or chromatin immunoprecipitation analyses, will allow for the determination of genes regulated by PPARγ in the ovary. The role of PPARα in ovarian steroidogenesis also needs to be better understood. Although PPARα null-mutant mice seem to reproduce normally, because activation of this isotype, as well as PPARγ, by exogenous agents alters ovarian steroid production, it may be a player and/or have a role in orchestrating ovarian hormone production. Because PPARδ can negatively regulate the activity of the other PPARs and is co-expressed in ovarian cells with PPARs α and γ, how this isotype my modulate the activity of PPARα and/or γ needs to be determined. Altering the ratio of PPARδ to PPARγ and/or PPARα within ovarian cells and how this affects the activity of the latter PPAR isotypes will add to the knowledge of how these transcription factors are regulated in the ovary. Also, understanding what triggers the expression of the PPARs in the ovary will further elucidate how gene expression in the ovary is regulated to support its normal, cyclic function.

## Conclusion

**Figure 5:**
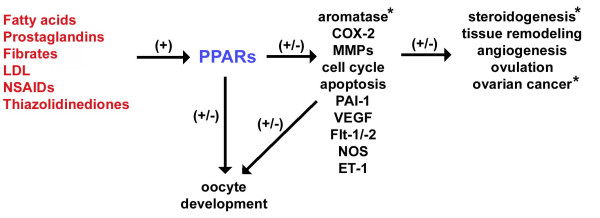
Proposed mechanisms by which PPARs may impact ovarian function and female fertility. The flow chart illustrates the potential interactions between the activation of PPARs and various factors known to impact processes critical for normal ovarian function. See text for details. Stimulatory impact is indicated by a (+). The ability to both stimulate and/or inhibit is denoted by (+/-). COX-2 = cyclooxygenase 2; ET-1 = endothelin -1; LDL = low density lipoprotein; MMPs = matrix metalloproteinases; NOS = nitric oxide synthase; NSAIDs = non-steroidal anti-inflammatory drugs; PAI-1 = plasminogen activator inhibitor -1; VEGF = vascular endothelial growth factor. Asterisk (*) denotes reported targets of PPARs in the ovary.

There are a variety of mechanisms by which PPARs could potentially influence ovarian function, as illustrated in Figure 5. The steady expression pattern of PPARs α and δ in the ovary during follicular development and the periovulatory period suggest that these PPAR isotypes may regulate gene expression involved in basal functioning of ovarian cells under normal physiological conditions. The ability of PPARγ to regulate ovarian function has been illustrated by agonists regulating steroid production by ovarian cells *in vitro*, and the sub- or infertility observed in animals with PPARγ disrupted in the ovary. The ability of metabolic factors (i.e. fatty acids) to activate PPARs allows for these transcription factors to alter gene expression in response to the nutritional status of the animal. Therefore, PPARs can mediate the influence of nutrition on female fertility. In addition, environmental exposure to agents such as phthalates and polycyclic aromatic hydrocarbons can also influence gene transcription through the PPARs.

The importance of understanding of the role(s) of PPARs in the ovary is indicated by their identification in healthy tissue, and altered expression in pathological ovarian tissues. Manipulation of these transcription factors could prove to be beneficial in either the treatment of ovarian pathologies, or as a means to regulate/improve fertility. As more is learned about the impact of PPARs on ovarian function, it will advance our understanding of the pattern of gene expression driving normal ovarian function, what goes awry leading to its dysfunction, and the role of these factors in mediating nutritional and environmental impacts on female fertility.
